# High blood pressure-targeted management associated with reduced benefits of higher body mass index in elderly patients with septic shock: a post hoc analysis of a multicenter randomized controlled trial

**DOI:** 10.1016/j.aicoj.2026.100157

**Published:** 2026-09-14

**Authors:** Takashi Shimazui, Tadanaga Shimada, Takehiko Oami, Akira Endo, Kazuma Yamakawa, Takashi Tagami, Yutaka Umemura, Keiichiro Shimoyama, Taka-aki Nakada

**Affiliations:** a Chiba University Graduate School of Medicine, Department of Emergency and Critical Care Medicine, 1-8-1 Inohana, Chuo-ku, Chiba, Japan; b Department of Acute Critical Care Medicine, Tsuchiura Kyodo General Hospital, 4-1-1 Otsuno, Tsuchiura, Ibaraki, Japan; c Department of Anesthesiology and Critical Care Medicine, Division of Acute and Critical Care Medicine, Hokkaido University Faculty of Medicine, Nishi 7, Kita-ku, Sapporo, Hokkaido, Japan; d Department of Emergency and Critical Care Medicine, Osaka Medical and Pharmaceutical University, 2‑7 Daigakumachi, Takatsuki, Osaka, Japan; e Department of Emergency and Disaster Medicine, The Jikei University School of Medicine, 3-25-8 Nishi-Shimbashi, Minato-ku, Tokyo, Japan; f Division of Trauma and Surgical Critical Care, Osaka General Medical Center, 3‑1‑56 Bandai‑Higashi, Sumiyoshi, Osaka, Japan; g Department of Neurointensive Care and Coma Science Center, Toda Medical Group, Asaka Medical Center, 1340-1, Mizonuma Asaka, Saitama, Japan

**Keywords:** Arterial pressure, Body mass index, Obesity, Septic shock, Vasoconstrictor agents

## Abstract

**Background:**

Body mass index (BMI) is an important prognostic factor in sepsis, and an obesity paradox has been reported. A higher mean arterial pressure (MAP) target has been associated with worse outcomes in elderly patients with septic shock. However, whether MAP target strategy alters the association between BMI and outcomes in this population is unknown.

**Methods:**

We conducted a post hoc analysis of the OPTPRESS trial, a multicenter randomized controlled trial at 29 Japanese centers reporting worse outcomes with a high MAP target in elderly patients with septic shock. A total of 489 patients aged ≥65 years with septic shock were assigned to a control MAP target of 65–70 mmHg (n = 245) or high target of 80–85 mmHg (n = 244). Patients were categorized using Asian BMI cutoffs into underweight (<18.5 kg/m^2^), normal-weight (18.5–22.9 kg/m^2^), and higher-BMI (≥23 kg/m^2^) categories. The primary outcome was 90-day mortality. The primary analysis assessed the interaction between BMI category and MAP target strategy.

**Results:**

In the control group, mortality decreased across increasing BMI categories (P = 0.03 for the trend), while no trend was observed in the high-target group (P = 0.26 for the trend). After adjustment for age, sex, APACHE II score, Clinical Frailty Scale, and site of infection, MAP target strategy significantly interacted with the higher-BMI category (P = 0.02), but not with underweight (P = 0.41). In the high-target group, both underweight and higher-BMI patients had higher mortality than those with normal BMI (underweight, adjusted HR 1.74 [95% CI 1.02–2.98], P = 0.04; higher BMI, adjusted HR 1.72 [95% CI 1.02–2.87], P = 0.04). Fluid volume per body weight decreased across increasing BMI categories in both groups. The proportion of patients receiving vasopressin increased across BMI categories only in the control group, whereas the body weight-adjusted vasopressin dose among recipients showed no significant trend in either group.

**Conclusions:**

In elderly patients with septic shock, mortality decreased across increasing BMI categories under a standard MAP target, whereas patients in the higher-BMI category had higher mortality under a high MAP target. High MAP targets may attenuate the survival benefit of higher BMI, possibly in association with BMI-related patterns in fluid and vasopressor management.

**Trial registration:**

Not registered. The original OPTPRESS trial was registered at UMIN Clinical Trials Registry (UMIN000041775) on 13 September 2020.

## Background

Sepsis is a life-threatening condition that accounts for approximately 20% of deaths worldwide [1]. Despite recent advances in critical care, no specific therapy has been established beyond antimicrobial treatments and organ-supportive measures, including fluid management and vasopressor use. Because circulatory failure contributes to multiple organ dysfunction and mortality [2], higher blood pressure targets have traditionally been hypothesized to improve organ perfusion and outcomes. However, a randomized controlled trial (RCT) in adults with septic shock showed no mortality benefit from a higher blood pressure target [3], and a recent RCT in elderly patients was terminated early because a higher mean arterial pressure (MAP) target was associated with increased mortality [4]. Given these findings, higher blood pressure targets may not be uniformly beneficial, underscoring the importance of identifying factors associated with vulnerability to such management for more individualized blood pressure targets.

Body mass index (BMI) is an important determinant of outcomes in critical illnesses, including sepsis [[5], [6], [7], [8]]. A Japanese study reported that underweight was associated with increased mortality in patients with sepsis [5], whereas a South Korean study found that obesity was associated with lower mortality and better functional outcomes [6]. This paradoxical survival benefit in obese patients, known as the obesity paradox [[7], [8], [9]], may also be observed in elderly patients with sepsis [10]. While the underlying mechanisms are unclear, proposed mechanisms include greater metabolic reserves and potential anti-inflammatory or immunomodulatory effects of adipose tissue. However, obese patients may face a greater cardiac workload through systemic and pulmonary vascular mechanisms, including elevated peripheral vascular resistance, aortic stiffness, and obesity-related respiratory disorders [11,12]. Vasopressors act on the cardiovascular system, and the hemodynamic effects of targeting a higher blood pressure may consequently differ according to BMI. Despite these considerations, whether body mass influences the association between blood pressure management and outcomes in sepsis remains insufficiently investigated.

In this study, we explored whether the association between BMI and clinical outcomes in sepsis differs according to blood pressure management. To elucidate these associations, we conducted a post hoc analysis of an RCT performed in Japan that compared different blood pressure targets in elderly patients with septic shock.

## Methods

### Study setting and patients

This study retrospectively analyzed patients enrolled in the multicenter, pragmatic, open-label RCT conducted at 29 Japanese centers (OPTPRESS trial) [4]. As this was an exploratory post hoc analysis, the sample size was determined by the original OPTPRESS trial. Patients were excluded if their BMI was not recorded. The detailed protocol of the OPTPRESS trial has been published elsewhere [13]. Briefly, this study enrolled patients aged ≥65 years with septic shock and randomized them into two groups with different mean arterial pressure (MAP) targets; a control group (target MAP 65–70 mmHg) and a high-target group (target MAP 80–85 mmHg). Initial vasopressor management was protocolized; if a norepinephrine dose ≥0.1 μg/kg/min was required to achieve the target MAP, vasopressin was initiated and titrated up to 0.04 U/min. If the MAP remained below target, the treating physician could add another vasopressor, administer hydrocortisone, or increase norepinephrine. Fluid administration and decisions to reduce or discontinue vasopressors, as well as other sepsis treatments, were at the treating physician’s discretion.

The OPTPRESS trial was approved by local ethics committees or institutional review boards. This post hoc retrospective analysis was approved by the Chiba University Hospital Certified Clinical Research Review Board (No. M10948). Written informed consent was obtained in the original trial, whereas it was waived for this retrospective study because of the study design. The study followed the STROBE guidelines [14].

### Data collection and definition

Baseline characteristics including age, sex, body weight (BW), BMI, Clinical Frailty Scale (CFS), comorbidities, source of infection, and severity scores (Acute Physiology and Chronic Health Evaluation [APACHE II] score, Sequential Organ Failure Assessment [SOFA] score) were retrieved. Treatments to maintain MAP during the first 72 h, such as fluid volume and doses of norepinephrine, vasopressin, and epinephrine, along with achieved MAP values at baseline and every 4 h through 72 h and the time required to achieve the assigned MAP target, were also retrieved. Norepinephrine doses were reported as norepinephrine base equivalents, consistent with recent recommendations on norepinephrine formulation reporting [15]. Outcome measures included 90-day mortality after randomization, 28-day organ support-free days, and adverse events (any arrhythmia, ischemic events, and hemorrhagic events). Organ support–free days were defined as the number of days from day 1 through day 28 after randomization during which patients were alive and free from the respective support for at least 24 consecutive hours. Patients who died within 28 days or remained on the respective support on day 28 were assigned zero organ support–free days. Vasopressor support included catecholamines and vasopressin. Missing data were not imputed as the amount was minimal (APACHE II, n = 1; 72 -h fluid and vasopressor treatment data, n = 7; 28-day organ support-free days, n = 1; safety endpoints, n = 4). The longitudinal MAP analysis included all available measurements from 4 to 72 h without imputing values unavailable after death or discontinuation of monitoring.

Patients in each control and high-target group were categorized into three groups based on their BMI: underweight (BMI < 18.5 kg/m^2^), normal weight (BMI 18.5–22.9 kg/m^2^), and higher BMI (BMI ≥ 23 kg/m^2^). Because the original OPTPRESS trial was conducted in Japan and most patients included were Japanese (506/516, 98.1%) [4], BMI cutoffs specific to the Asian population were used for categorization [16]. As only 16 of 489 patients (3.3%) had a BMI ≥ 30 kg/m^2^, the World Health Organization threshold for obesity, a sensitivity analysis using a BMI cutoff of ≥25 kg/m^2^, the threshold for overweight, was also examined [17].

### Statistical analysis

The primary outcome was all-cause 90-day mortality; all patients were followed for up to 90 days or until death. Secondary outcomes included 28-day ventilator-free days, renal replacement therapy (RRT)-free days, vasopressor-free days, and adverse events, which were evaluated as safety endpoints in the original trial [4].

The primary analysis assessed the interaction between BMI category and MAP target strategy for 90-day mortality. Cox regression models were fitted with MAP target group, BMI category, and their interaction term. Multivariable models were adjusted for age, sex, APACHE II score, CFS, and site of infection to account for potential imbalances in baseline characteristics. These covariates were selected based on their clinically plausible prognostic relevance [18,19], while maintaining model parsimony given the number of outcome events. Stratified Cox regression analysis was also performed within each MAP target group to estimate hazard ratios (HRs) with 95% confidence intervals (CIs) for 90-day mortality according to BMI category.

To support the primary analysis, differences across BMI categories within each MAP target group were examined, with P values interpreted descriptively. Trends across BMI categories were evaluated using the Cochran–Armitage trend test for categorical variables and the Jonckheere–Terpstra test for continuous variables. Between-group differences were evaluated using the Wilcoxon rank-sum test or Kruskal–Wallis test for continuous variables and Pearson’s chi-square test for categorical variables. Survival curves were compared using the log-rank test or log-rank test for trend, as appropriate. Achieved MAP after randomization (4–72 h) was compared across BMI categories within each MAP target group using a mixed-effects model. Sensitivity analyses included using an alternative cutoff of ≥25 kg/m^2^ for the higher-BMI category, further adjusting the interaction and MAP-stratified Cox models for chronic heart failure, chronic kidney disease, diabetes, and chronic hypertension, and modeling BMI as a continuous variable using restricted cubic splines. The spline analysis used a Cox proportional hazards model adjusted for age, sex, APACHE II score, CFS, and site of infection and included an interaction between BMI spline terms and MAP target strategy. The number of knots was selected based on the lowest Akaike information criterion among models with three, four, and five knots. The selected three-knot model placed knots at the 10th, 50th, and 90th percentiles. Likelihood-ratio tests assessed group-specific nonlinearity and the overall interaction, and adjusted HRs with profile-likelihood 95% CIs were estimated relative to the cohort median BMI of 21.4 kg/m^2^ within each MAP target group.

Data are expressed as medians with interquartile ranges (IQR) for continuous variables and as absolute numbers with percentages for categorical variables. Statistical significance was defined as a two-tailed p < 0.05. Analyses were performed with JMP Student Edition 18.2.2 software (SAS Institute Inc., Cary, NC, USA), GraphPad Prism 10.4.1 (GraphPad Software, Boston, MA, USA) for mixed-effects models, and Python 3.11.3 with lifelines 0.30.3, statsmodels 0.14.6, and SciPy 1.17.1 for restricted cubic spline analyses.

## Results

A total of 489 patients (245 in the control group and 244 in the high-target group) were analyzed (Supplementary Figure S1). Among the patients in the control group, 65 were categorized as underweight, 88 as normal weight, and 92 as higher BMI; among those in the high-target group, 61 were categorized as underweight, 105 as normal weight, and 78 as higher BMI (Table 1). APACHE II and SOFA scores were similar across BMI categories within each MAP target group, indicating comparable severity (APACHE II: control, P = 0.43; high-target, P = 0.78; SOFA: control, P = 0.75; high-target, P = 0.31).

**Table 1:** Baseline characteristics in each group.

	Control group			High-target group		
	Underweight (n = 65)	Normal (n = 88)	Higher BMI (n = 92)	Underweight (n = 61)	Normal (n = 105)	Higher BMI (n = 78)
Age, yr	82 (74–87)	77 (72–85)	77 (72–83)	79 (73–84)	79 (75–85)	79 (73–84)
Male sex, n (%)	30 (46.2)	55 (62.5)	47 (51.1)	34 (55.7)	66 (62.9)	40 (51.3)
Body mass index	16.6 (15.5–17.6)	20.9 (19.9–22.0)	25.5 (24.3–28.3)	16.7 (15.6–17.4)	21.0 (19.8–22.2)	25.9 (24.8–28.2)
Clinical frailty scale	5 (4–6)	4 (3–6)	4 (3–6)	5 (3–7)	5 (3–6)	4 (3–5)
Comorbidities, n (%)						
Chronic hypertension	29 (44.6)	48 (54.5)	60 (65.2)	26 (42.6)	51 (48.6)	57 (73.1)
Ischemic heart disease	4 (6.2)	10 (11.4)	10 (10.9)	10 (16.4)	10 (9.5)	9 (11.5)
Chronic heart failure	15 (23.1)	20 (22.7)	8 (8.7)	10 (16.4)	13 (12.4)	23 (29.5)
COPD	2 (3.1)	3 (3.4)	5 (5.4)	5 (8.2)	4 (3.8)	3 (3.8)
Chronic kidney disease	6 (9.2)	11 (12.5)	19 (20.7)	8 (13.1)	16 (15.2)	16 (20.5)
Liver cirrhosis	0 (0.0)	2 (2.3)	4 (4.3)	2 (3.3)	1 (1.0)	2 (2.6)
Diabetes mellitus	11 (16.9)	27 (30.7)	35 (38.0)	15 (24.6)	25 (23.8)	24 (30.8)
Cancer	10 (15.4)	9 (10.2)	14 (15.2)	15 (24.6)	21 (20.0)	7 (9.0)
Source of infection						
Lung	22 (33.8)	22 (25.0)	17 (18.5)	31 (50.8)	24 (22.9)	7 (9.0)
Abdomen	15 (23.1)	26 (29.5)	32 (34.8)	13 (21.3)	31 (29.5)	33 (42.3)
Urinary tract	20 (30.8)	26 (29.5)	27 (29.3)	9 (14.8)	27 (25.7)	24 (30.8)
Soft tissue	3 (4.6)	15 (17.0)	9 (9.8)	4 (6.6)	8 (7.6)	8 (10.3)
Blood	3 (4.6)	1 (1.1)	4 (4.3)	1 (1.6)	6 (5.7)	2 (2.6)
Other	1 (1.5)	1 (1.1)	2 (2.2)	1 (1.6)	1 (1.0)	1 (1.3)
Unknown	1 (1.5)	2 (2.3)	8 (8.7)	5 (8.2)	12 (11.4)	6 (7.7)
APACHE II score	26 (21–33)	24 (20–29)	26 (21–32)	27 (22–32)	27 (20–33)	26 (21–31)
SOFA score	10 (7–11)	9 (7–12)	10 (8–12)	9 (7–12)	11 (8–12)	10 (8–12)

COPD, chronic obstructive pulmonary disease; APACHE, Acute Physiology and Chronic Health Evaluation; SOFA, Sequential Organ Failure Assessment. Values are expressed as median (interquartile range).

### Association of BMI category with mortality according to MAP target strategy

In the control group, mortality showed a significant decreasing trend from the underweight to the higher-BMI category (underweight, 36.9%; normal weight, 26.1%; higher BMI, 20.7%; P = 0.03 for the trend). In the high-target group, underweight patients showed the highest mortality, whereas patients in the higher-BMI category also had relatively high mortality, with no trend observed across BMI categories (underweight, 49.2%; normal weight, 30.5%; higher BMI, 38.5%; P = 0.26 for the trend) (Fig. 1).

**Fig. 1:**
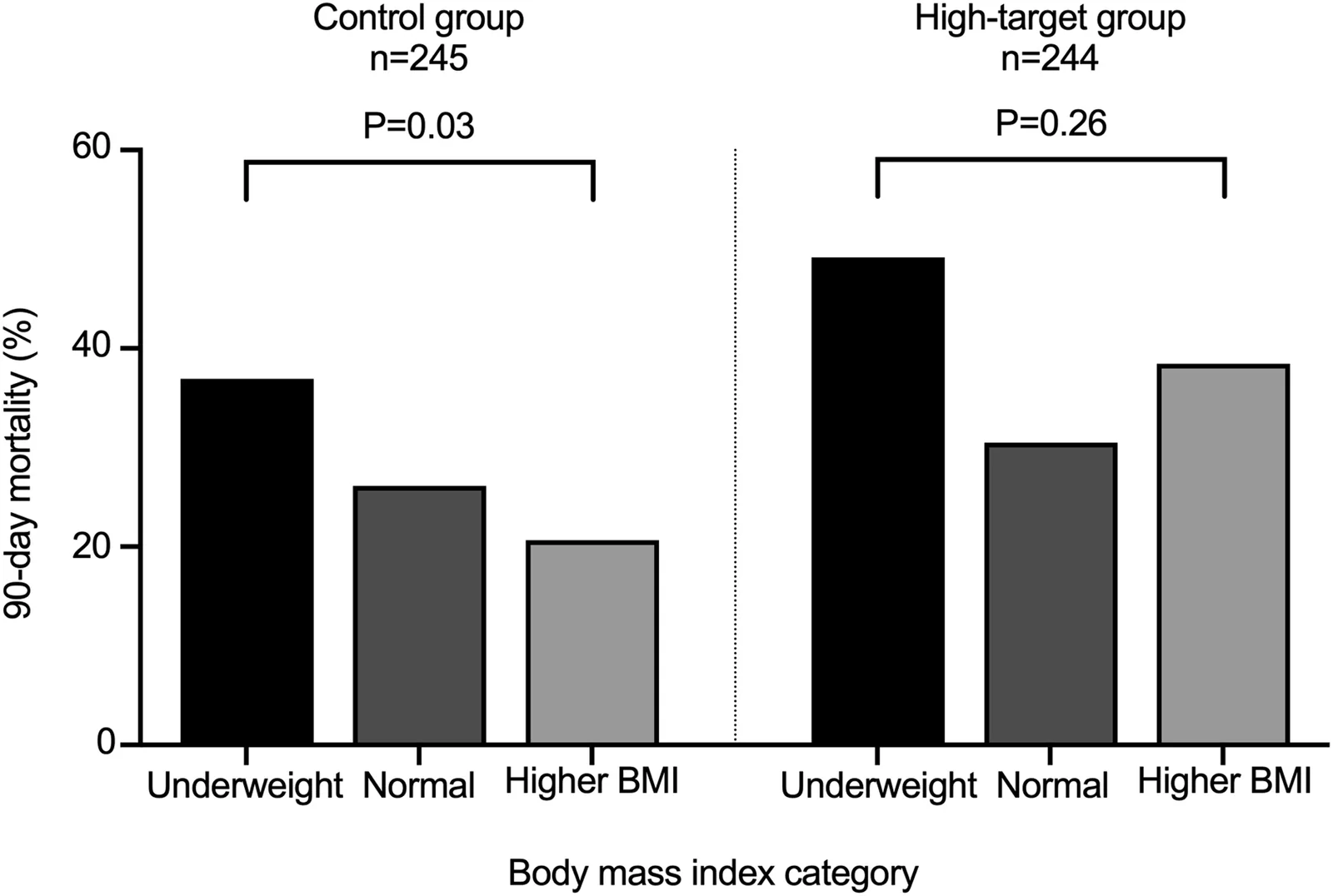
90-day mortality in each group according to the body mass index categories. Patients in the control group showed a significant inverse trend in mortality across increasing BMI categories (P = 0.03 for the trend). Patients in the high-target group had the highest mortality among underweight patients, and higher-BMI patients also exhibited high mortality, with no significant trend across BMI categories (P = 0.26 for the trend). P-values were calculated with Cochran–Armitage test for trend.

Using normal weight as the reference, the unadjusted interaction model showed no significant interactions between MAP target strategy and underweight (P = 0.64) or higher BMI (P = 0.15) for mortality. However, after adjustment for baseline factors, a significant interaction was observed between MAP target strategy and higher BMI (adjusted HR [95% CI], 2.63 [1.18–5.82], P = 0.02), whereas the interaction with underweight was not significant (P = 0.41) (Supplementary Table S1). The interaction remained significant even after additional adjustment for the comorbidities (P = 0.02) (Supplementary Table S2). Restricted cubic spline analysis showed no evidence of nonlinearity in the BMI–mortality association in the control group (P = 0.75), whereas a significant nonlinear association was observed in the high-target group (P = 0.007). There was a significant overall interaction between BMI spline terms and MAP target strategy (P = 0.047) (Fig. 2).

**Fig. 2:**
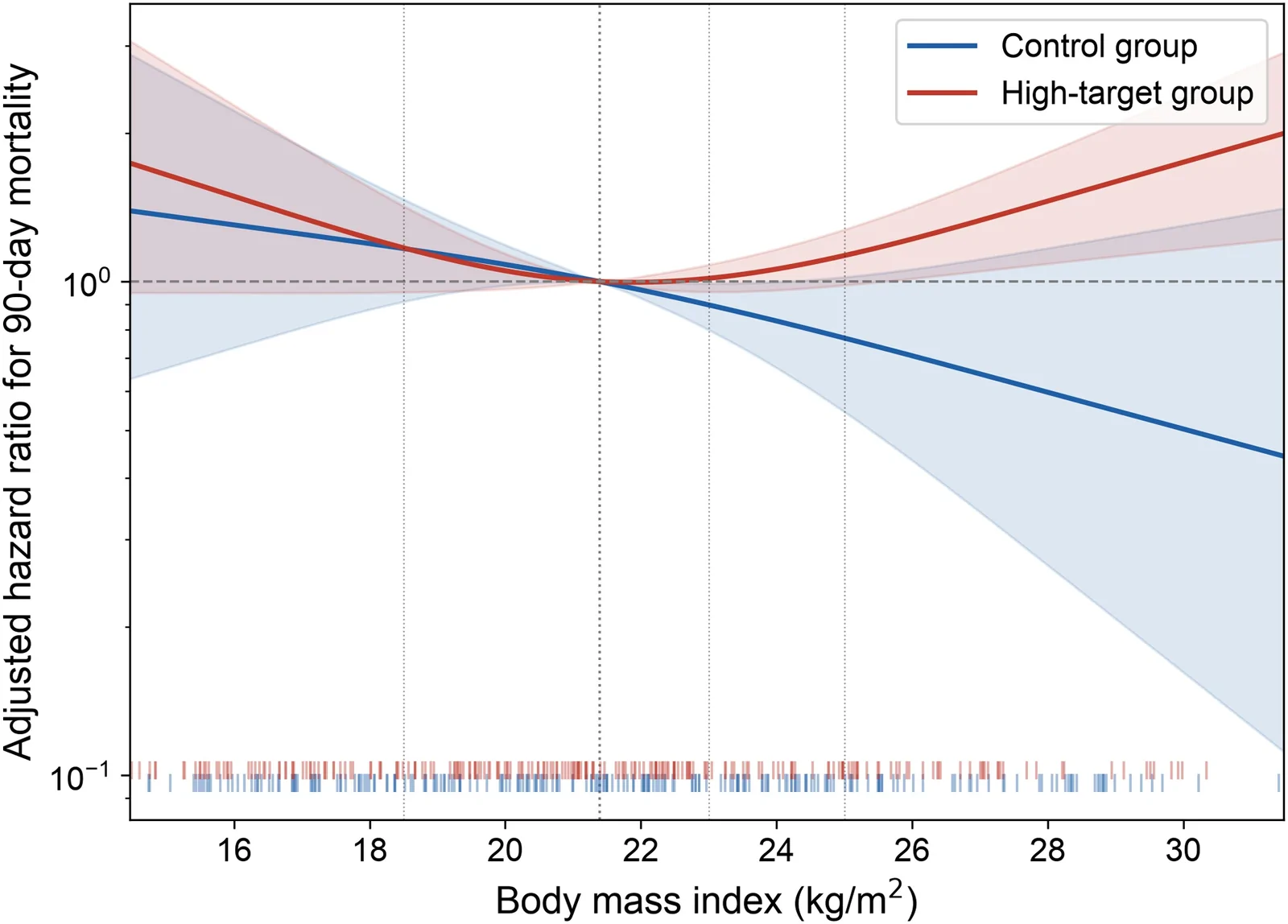
Adjusted spline associations of body mass index with mortality by mean arterial pressure target. Restricted cubic splines were fitted with three knots at the 10th, 50th, and 90th percentiles. Solid lines and shaded areas indicate adjusted hazard ratios and pointwise profile-likelihood 95% confidence intervals, respectively, using the cohort median BMI of 21.4 kg/m^2^ as the within-group reference (the darker dotted line). The model was adjusted for age, sex, APACHE II score, CFS, and site of infection. Vertical dotted lines indicate BMI values of 18.5, 23, and 25 kg/m^2^; rug plots show the BMI distributions. Nonlinearity: control group, P = 0.75; high-target group, P = 0.007. Overall interaction: P = 0.047.

Stratified Cox regression analysis within each MAP target group showed that underweight was associated with an increased risk of 90-day mortality in both treatment arms, reaching statistical significance in the high-target group but not in the control group (control, P = 0.18; high-target, P = 0.03). The higher-BMI category was not significantly associated with mortality in either treatment arm, although the direction of association differed between the control and high-target groups (control, HR [95% CI] 0.76 [0.42–1.40], P = 0.39; high-target, HR [95% CI] 1.36 [0.82–2.23], P = 0.23) (Supplementary Table S3A). These associations persisted after adjustment for baseline covariates. Specifically, patients in the higher-BMI category in the high-target group exhibited an adjusted HR for mortality similar to that of underweight patients (adjusted HR [95% CI] for underweight in the high-target, 1.74 [1.02–2.98], P = 0.04; for higher BMI in the high-target, 1.72 [1.02–2.87], P = 0.04) (Supplementary Table S3B). Stratified analyses with additional adjustment for the specified comorbidities yielded similar results (Supplementary Table S4).

Survival-curve analysis showed a significant decreasing trend in mortality from the underweight to the higher-BMI category in the control group (P = 0.03 for the trend), whereas no such trend was seen in the high-target group (P = 0.83 for the trend) (Supplementary Figure S2). Comparison of the survival curves between the control and high-target groups within each BMI category revealed a significant difference only in the higher-BMI category (P = 0.008), while no significant difference was observed in the underweight category (P = 0.15) (Fig. 3).

**Fig. 3:**
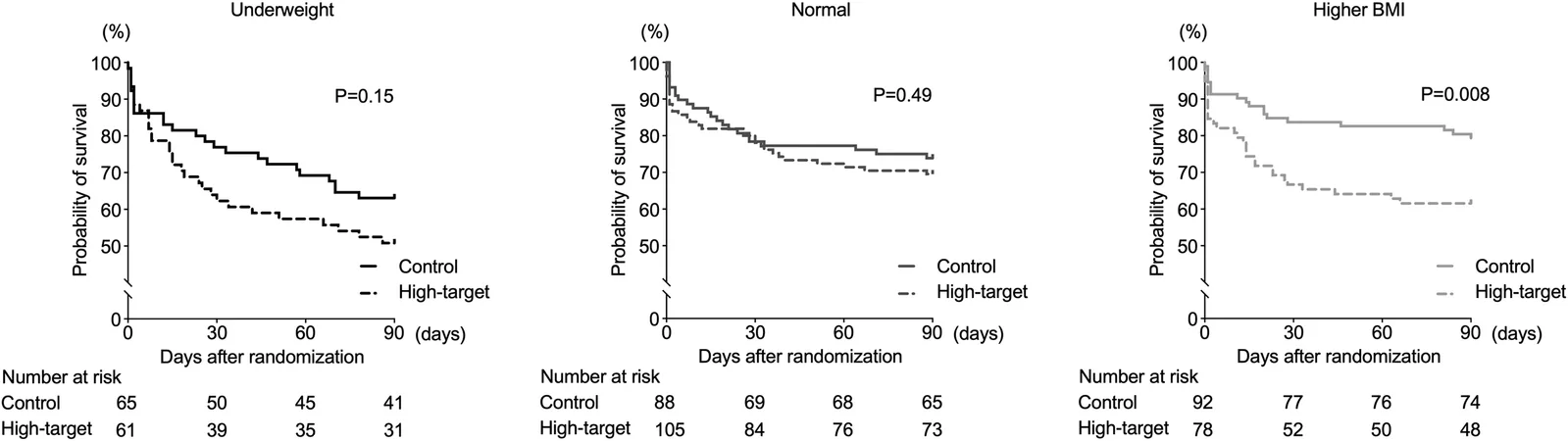
Survival curves between the control and high-target groups in each body mass index category. Higher-BMI patients showed a significant difference in mortality between control and high-target groups (P = 0.008), while nonsignificant differences were observed in the underweight (P = 0.15) and normal weight (P = 0.49) categories. P-values were calculated with Log-rank test

A sensitivity analysis using a cutoff of ≥25 kg/m^2^ for the higher-BMI category yielded similar findings. Mortality showed a decreasing trend across increasing BMI categories in the control group but not in the high-target group (Supplementary Figure S3). The interaction between BMI ≥ 25 kg/m^2^ and MAP target strategy was not significant before adjustment (P = 0.18) but was significant after adjustment (P = 0.03). Stratified Cox and survival analyses were also consistent with the primary analysis (Supplementary Table S5 and Supplementary Figure S4).

### Effect of blood pressure targets on secondary outcomes and adverse events by BMI

In exploratory analyses evaluating the impact of blood pressure management on secondary outcomes across BMI categories, organ support–free days were generally fewer in the high-target group among underweight patients and those in the higher-BMI category, whereas no clear differences were observed among patients with normal BMI (Table 2). With respect to blood pressure- and vasopressor-related adverse events, ischemic events were numerically more frequent among patients in the higher-BMI category in the high-target group (P = 0.08), and hemorrhagic events were numerically more frequent among patients with normal BMI in the high-target group (P = 0.06), although neither reached nominal statistical significance (Table 2). No clear ordered differences in secondary outcomes or adverse events were observed across BMI categories within each treatment arm (Supplementary Table S6).

**Table 2:** Secondary outcomes and adverse events between blood pressure target groups by body mass index category.

	Control group	High-target group	P-value
28-day ventilator-free days			
Underweight	28 (0–28)	15 (0–27)	0.006
Normal	22 (0–28)	23 (0–28)	0.99
Higher BMI	25 (16–28)	21 (0–28)	0.05
28-day RRT-free days			
Underweight	28 (0–28)	28 (0–28)	0.08
Normal	28 (11–28)	28 (8–28)	0.85
Higher BMI	28 (25–28)	28 (0–28)	0.02
28-day vasopressor-free days			
Underweight	25 (17–26)	22 (0–25)	0.03
Normal	24 (18–25)	24 (19–25)	0.55
Higher BMI	25 (20–26)	23 (0–25)	<0.001
Any arrhythmia, n (%)			
Underweight	5 (7.7)	9 (14.8)	0.21
Normal	10 (11.4)	12 (11.4)	0.99
Higher BMI	9 (9.8)	7 (9.0)	0.86
Ischemic events, n (%)			
Underweight	4 (6.2)	3 (4.9)	0.76
Normal	1 (1.1)	5 (4.8)	0.15
Higher BMI	4 (4.3)	9 (11.5)	0.08
Hemorrhagic events, n (%)			
Underweight	1 (1.5)	1 (1.6)	0.96
Normal	0 (0.0)	4 (3.8)	0.06
Higher BMI	2 (2.2)	3 (3.8)	0.52

RRT, renal replacement therapy.

Values are expressed as median (interquartile range). P-values were calculated using Wilcoxon rank sum test or Pearson’s chi-square test.

### Achieved MAP and treatment requirements across BMI categories

After randomization, achieved MAP did not differ significantly across BMI categories in either treatment arm, with no significant BMI category effect or BMI category-by-time interaction (control: P = 0.38 and P = 0.17; high-target: P = 0.83 and P = 0.32; Supplementary Figure S5). The time required to achieve the assigned MAP target also did not differ significantly across BMI categories in either arm (control: underweight, 1.00 [0.75–3.00] hours; normal, 1.00 [0.00–2.00] hours; higher BMI, 1.50 [0.00–3.00] hours; P = 0.11; high-target: underweight, 3.00 [1.00–6.00] hours; normal, 3.00 [1.00–8.00] hours; higher BMI, 4.00 [2.00–7.00] hours; P = 0.18).

Regarding treatments required to maintain blood pressure, total vasopressin dose showed a significant increasing trend from the underweight to the higher-BMI category in the control group (P = 0.01 for the trend), whereas no such trend was observed in the high-target group (P = 0.71 for the trend) (Supplementary Table S7). When the proportion of patients receiving vasopressin was further examined, it increased across BMI categories only in the control group (underweight, 34/64 [53.1%]; normal weight, 60/87 [69.0%]; higher BMI, 68/91 [74.7%]; P = 0.006 for the trend), with no trend in the high-target group (underweight, 50/60 [83.3%]; normal weight, 91/105 [86.7%]; higher BMI, 63/75 [84.0%]; P = 0.95 for the trend). No clear differences were observed in total fluid volume or the total doses of other vasopressors (Supplementary Table S7). When adjusted for BW, fluid volume showed a significant decreasing trend from the underweight to the higher-BMI category in both treatment arms (control, P < 0.001 for the trend; high-target, P < 0.001 for the trend), whereas weight-adjusted 72 -h vasopressor doses did not differ across BMI categories in either arm (Supplementary Table S7). Additionally, among vasopressin recipients, the BW-adjusted vasopressin dose showed no significant trend across BMI categories in either group (control: underweight, 0.74 [0.31–1.49]; normal weight, 0.55 [0.23–1.32]; higher BMI, 0.46 [0.18–0.93]; P = 0.09 for the trend; high-target: underweight, 0.99 [0.54–1.90]; normal weight, 0.79 [0.32–1.53]; higher BMI, 0.81 [0.30–1.38]; P = 0.12 for the trend).

## Discussion

In the present study, we observed a potential interaction between the higher-BMI category and MAP target strategy for mortality. The control group showed the obesity paradox, whereas both underweight and higher-BMI patients had high mortality in the high-target group. No interaction was observed between underweight and MAP target strategy. Consistent with these findings, organ support–free days were fewer in underweight and higher-BMI patients managed with the high-target strategy. Higher-BMI patients in the high-target group showed a trend toward more ischemic events. Fluid volume per BW decreased across increasing BMI categories in both arms. Although BW-adjusted vasopressor doses did not differ across BMI categories, the proportion of patients receiving vasopressin increased across BMI categories only in the control group.

Our study found that the association between the higher-BMI category and mortality differed according to MAP target strategy. A significant interaction emerged after adjustment for clinically selected prognostic factors, suggesting attenuation in the unadjusted model. Similar results were obtained after additional adjustment for major comorbidities. Consistently, exploratory spline analysis showed a significant overall interaction between BMI spline terms and MAP target strategy, with a nonlinear BMI–mortality association in the high-target group but no evidence of nonlinearity in the control group. The original OPTPRESS trial was terminated early because the high-MAP target strategy was associated with increased mortality [4]. Our findings extend this observation by suggesting that the adverse association of high MAP management may be particularly evident in patients in the higher-BMI category. Achieved MAP and time to target were similar across BMI categories, making inadequate MAP achievement unlikely to explain the higher mortality among higher-BMI patients in the high-target arm. Differences in fluid and vasopressor exposure may instead partly explain this finding. Although the causal relationships between high-dose norepinephrine, vasopressin use, and clinical outcomes have not been fully established, several studies report that the addition of vasopressin reduces norepinephrine requirements and may be associated with improved outcomes in certain cohorts [[20], [21], [22]]. Notably, a recent study using a reinforcement learning model suggested that initiating vasopressin at a lower norepinephrine dose (median 0.20 vs 0.37 μg/kg/min under standard care) was associated with reduced mortality [22]. A higher norepinephrine-equivalent dose at vasopressin initiation has also been associated with increased in-hospital mortality [23], and earlier vasopressin initiation at lower norepinephrine doses has been associated with better outcomes [22]. However, the catecholamine-sparing effect of vasopressin may vary according to BW [24]. In patients with septic shock, norepinephrine requirements increased after vasopressin initiation in obese patients but decreased in normal-weight patients, suggesting an attenuated hemodynamic response to vasopressin in obesity [25]. Obesity-related alterations in adrenergic signaling have also been reported, including reduced β3-adrenergic receptor expression in adipose tissue [26,27]. Collectively, these findings suggest that vasopressor responsiveness may differ according to BMI and potentially modify the association between MAP target strategy and mortality.

Vasopressor patterns observed in our cohort may provide further insight into the BMI–MAP target interaction. Total vasopressin dose increased across BMI categories only in the control group, primarily reflecting an increasing proportion of patients receiving vasopressin rather than higher BW-adjusted doses among recipients. In the OPTPRESS trial, vasopressin was initiated according to protocol when a norepinephrine dose of ≥0.1 μg/kg/min was required to achieve the target MAP [4,13]. Thus, patients with higher BMI in the control group more frequently reached the protocol-defined condition for vasopressin initiation, whereas in the high-target group vasopressin was required in most patients regardless of BMI category. Whether these patterns reflect differences in hemodynamic responsiveness cannot be determined, and a ceiling effect in the high-target group is an alternative explanation that the present data cannot confirm. These trends were assessed within each MAP target group and do not by themselves establish a differential association with MAP target strategy. However, they raise the possibility that weight-indexed vasopressor titration does not result in uniform vasopressin exposure across BMI categories, which warrants further investigation.

Beyond differences in vasopressin use, fluid administration patterns across BMI categories also merit consideration. Although absolute fluid volumes did not differ significantly across BMI categories, fluid volume per BW decreased from the underweight to the higher-BMI category in both groups. While excessive fluid administration may exacerbate tissue edema and worsen outcomes [28], relative hypovolemia combined with high-dose vasopressors such as norepinephrine may also be harmful. A propensity score-matched study of patients with hemorrhagic shock demonstrated that high-dose norepinephrine use was associated with significantly lower fluid administration, and that both high-dose norepinephrine and low fluid resuscitation were independently associated with higher SOFA score at 24 h [29]. Adequate fluid administration is necessary for optimal outcomes, even though excessive fluid administration is detrimental. Moreover, excess body mass in obesity requires increases in intravascular blood volume and cardiac output to maintain adequate tissue perfusion [30]. However, whether relatively lower fluid administration in patients with higher BMI resulted in insufficient intravascular volume and subsequent tissue hypoperfusion could not be evaluated, as preload, fluid responsiveness, cardiac output, and tissue perfusion were not measured. Patients in the higher-BMI category in the high-target group showed a nonsignificant trend toward more ischemic events. Because the decrease in fluid volume per BW was observed in both MAP target groups, this finding alone is unlikely to explain the interaction between BMI category and MAP target strategy. However, in the high-target group, comparatively lower weight-indexed fluid administration coexisted with greater reliance on vasopressor support, and a contribution of this combination to outcomes cannot be excluded, meriting direct evaluation with hemodynamic and perfusion measurements.

The counterintuitive observation that patients with obesity experience better outcomes in certain diseases, a pattern also observed in our control group, is known as the obesity paradox [[7], [8], [9]]. This phenomenon has also been reported in sepsis, particularly among older patients, consistent with our findings. A previous meta-analysis showed that overweight and obesity were associated with lower mortality among patients aged >50 years, whereas this association was not significant among those aged ≤50 years [10]. Proposed explanations include the adverse prognostic effect of malnutrition, greater metabolic reserves that may improve tolerance to the catabolic state of critical illness, circulating lipids that bind endotoxins, and differences in neurohormonal activity [8,9]. Conversely, in line with previous reports in sepsis and other critical illnesses [5,10,31], underweight patients in our study had higher mortality regardless of blood pressure management strategy.

Our study analyzed a large, well-characterized cohort derived from a multicenter RCT, ensuring high-quality and systematically collected data, which represents a strength of this study. Additionally, the availability of detailed information on blood pressure management including fluid administration and vasopressor dosing as well as clinically relevant outcomes such as ischemic events allowed us to explore potential mechanistic insights into the associations between BMI and outcomes in sepsis. However, despite these strengths and the novel insights provided by this study, several limitations should be acknowledged. First, this was an exploratory post hoc analysis that was not prospectively planned, and its retrospective nature may have introduced selection, treatment, and misclassification bias. In addition, multiple exploratory analyses were performed, and secondary findings should therefore be interpreted cautiously as hypothesis-generating. Longitudinal MAP measurements were unavailable after death or discontinuation of monitoring, and the achieved-MAP analysis may therefore have been affected by missingness related to clinical course. Furthermore, subgroup analyses within each randomized treatment arm resulted in relatively small sample sizes for each BMI category, which may have led to unstable estimates and limited the robustness of the findings. Second, since the original study did not prespecify detailed protocols for fluid administration and vasopressor titration according to BW or BMI, unmeasured confounding related to hemodynamic management may have influenced the observed associations. In addition, although vasopressin initiation was protocolized when a norepinephrine dose of ≥0.1 μg/kg/min was required to achieve the target MAP, the actual patient-level norepinephrine dose and timing at initiation were unavailable, precluding assessment of how variation in vasopressin initiation influenced the findings. Furthermore, the proposed hemodynamic mechanisms could not be evaluated directly, as preload, fluid responsiveness, cardiac output, and tissue perfusion were not measured. Third, as this study was conducted in a cohort consisting predominantly of Japanese patients, the generalizability of our findings to other ethnic populations remains unclear, even though the sensitivity analysis using a BMI cutoff of ≥25 kg/m^2^ showed similar results. In addition, only a small number of patients met the conventional World Health Organization obesity criterion of BMI ≥30 kg/m^2^, precluding analysis using this threshold. Fourth, although the model was adjusted using a small, fixed set of prognostic and demographic factors selected on clinical grounds, the statistical significance of the interaction between the higher-BMI category and MAP target strategy varied with covariate adjustment. Model instability and residual confounding, including differences in comorbidity burden across BMI categories, cannot be excluded, and this finding should therefore be interpreted with caution. Additionally, since the original OPTPRESS trial included only elderly patients (≥65 years), the effects in younger patients also remain uninvestigated. Further studies are required to determine whether similar observations can be replicated in other background groups.

## Conclusions

Mortality decreased across increasing BMI categories in elderly patients with septic shock under a standard MAP target, whereas patients in the higher-BMI category had higher mortality under a high MAP target. The higher-BMI category and MAP target strategy showed a significant interaction after adjustment for baseline imbalances. Fluid volume per BW decreased across increasing BMI categories in both groups, whereas total vasopressin dose and the proportion of patients receiving vasopressin increased across BMI categories only under the standard strategy. Although exploratory and hypothesis-generating, these findings suggest that blood pressure management, including fluid and vasopressor use, may influence the survival benefit associated with higher BMI.

## Author contributions

TS (Shimazui) contributed to the study conception and design, statistical analysis, interpretation of the data, and drafting of the manuscript. TS (Shimada), TO and TN contributed to the interpretation of the data and revised the manuscript for important intellectual content. AE, KY, TT, and YU contributed to the data acquisition and revised the manuscript for important intellectual content. KS revised the manuscript for important intellectual content. All the authors have read and approved the final version of the manuscript.

## Consent for publication

Not applicable.

## Ethics approval and consent to participate

The OPTPRESS trial was approved by local ethics committees or institutional review boards. This post hoc retrospective analysis was approved by the Chiba University Hospital Certified Clinical Research Review Board (No. M10948). Written informed consent was obtained in the original trial, whereas it was waived for this retrospective study because of the study design.

## Declaration of Generative AI and AI-assisted technologies in the writing process

During the preparation of this work, the authors used GPT-5.5 and Claude Opus 4.8 to improve the language and readability of the manuscript. The authors reviewed and edited the output as needed and take full responsibility for the content of the published article.

## Funding

This post hoc analysis received no specific funding. The original OPTPRESS trial was funded by the Japanese Society for the Promotion of Science KAKENHI, as described in the original publication.

## Data availability

The datasets analyzed in the current study are available from the corresponding author upon reasonable request.

## Declaration of competing interest

TN is the CEO of Smart119 Inc. and owns stock. Smart119 Inc. played no role in this study. The other authors declare that they have no conflicts of interest.
