# Supplementary material for: High blood pressure-targeted management associated with reduced benefits of higher body mass index in elderly patients with septic shock: a post hoc analysis of a multicenter randomized controlled trial
Source: Ann Intensive Care. 2026 Sep 14;16:100157. doi: 10.1016/j.aicoj.2026.100157 (PMC13634275; doi:10.1016/j.aicoj.2026.100157)
Supplement: Supplementary file 1 [file mmc1.docx]

**Supplementary Material**

**High Blood Pressure-Targeted Management Associated With Reduced Benefits of Higher Body Mass Index in Elderly Patients With Septic Shock: A Post Hoc Analysis of a Multicenter Randomized Controlled Trial**

Takashi Shimazui, Tadanaga Shimada, Takehiko Oami, Akira Endo, Kazuma Yamakawa, Takashi Tagami, Yutaka Umemura, Keiichiro Shimoyama, Taka-aki Nakada

**Contents:**

**Tables:**

**Supplementary Table S1.** Cox regression analyses for the interaction between body mass index category and mean arterial pressure target strategy.

**Supplementary Table S2.** Comorbidity-adjusted Cox regression analysis of the interaction between body mass index category and mean arterial pressure target strategy.

**Supplementary Table S3.** Cox regression analysis to identify the associations between body mass index categories and mortality in the different blood pressure target groups.

**Supplementary Table S4.** Comorbidity-adjusted Cox regression analysis to identify the associations between body mass index categories and mortality in the different blood pressure target groups.

**Supplementary Table S5.** Cox regression analysis to identify the associations between body mass index categories with a cutoff of ≥25 kg/m² and mortality in the different blood pressure target groups.

**Supplementary Table S6.** Trends in the secondary outcomes and adverse events across body mass index categories within each blood pressure target group.

**Supplementary Table S7.** Trends in the management required to maintain blood pressure during the 72 hours after randomization across body mass index categories within each blood pressure target group.

**Figures:**

**Supplementary Figure S1.** Patient flow diagram.

**Supplementary Figure S2.** Survival curves between body mass index categories in the control and high-target groups.

**Supplementary Figure S3.** 90-day mortality in the control and high-target groups using a cutoff of ≥25 kg/m² to define the higher body mass index category.

**Supplementary Figure S4.** Survival curves in the control and high-target groups using ≥25 kg/m² to define the higher body mass index category.

**Supplementary Figure S5.** Blood pressure management according to mean arterial pressure target across different body mass index categories.

**Supplementary Table S1.** Cox regression analyses for the interaction between body mass index category and mean arterial pressure target strategy.

A. Unadjusted interaction model

|  | Hazard ratio (95% CI) | P-value |
| --- | --- | --- |
| Body mass index category |  |  |
| Underweight | 1.60 (1.10–2.35) | 0.01 |
| Normal | Reference |  |
| Higher BMI | 1.02 (0.69–1.51) | 0.92 |
| High-MAP target strategy | 1.77 (1.19–2.62) | 0.005 |
| Interaction with underweight | 1.20 (0.56–2.56) | 0.64 |
| Interaction with higher BMI | 1.78 (0.81–3.93) | 0.15 |

B. Adjusted interaction model

|  | Adjusted hazard ratio (95% CI) | P-value |
| --- | --- | --- |
| Body mass index category |  |  |
| Underweight | 1.53 (1.04–2.27) | 0.03 |
| Normal | Reference |  |
| Higher BMI | 1.08 (0.73–1.61) | 0.70 |
| High-MAP target strategy | 1.73 (1.17–2.57) | 0.007 |
| Interaction with underweight | 1.39 (0.64–3.01) | 0.41 |
| Interaction with higher BMI | 2.63 (1.18–5.82) | 0.02 |
| Age | 1.01 (0.98–1.03) | 0.54 |
| Male sex | 1.15 (0.82–1.63) | 0.42 |
| APACHE II score | 1.07 (1.05–1.09) | <0.001 |
| Clinical frailty score | 1.19 (1.08–1.33) | <0.001 |
| Site of infection |  |  |
| Lung | 1.11 (0.72–1.72) | 0.64 |
| Abdomen | 1.13 (0.74–1.73) | 0.56 |
| Urinary tract | 0.48 (0.29–0.79) | 0.004 |
| Others | Reference |  |

APACHE: Acute Physiology and Chronic Health Evaluation; MAP: mean arterial pressure.

Hazard ratio associated with a one-unit change of age, APACHE II score, and clinical frailty score. Interaction hazard ratios indicate how the effect of the high-MAP target strategy differs from that in the normal-body mass index group.

**Supplementary Table S2.** Comorbidity-adjusted Cox regression analysis of the interaction between body mass index category and mean arterial pressure target strategy.

|  | Adjusted hazard ratio (95% CI) | P-value |
| --- | --- | --- |
| Body mass index category |  |  |
| Underweight | 1.51 (1.02–2.24) | 0.04 |
| Normal | Reference |  |
| Higher BMI | 1.10 (0.73–1.65) | 0.65 |
| High-MAP target strategy | 1.73 (1.15–2.57) | 0.007 |
| Interaction with underweight | 1.47 (0.67–3.19) | 0.34 |
| Interaction with higher BMI | 2.68 (1.20–6.03) | 0.02 |
| Age | 1.01 (0.98–1.03) | 0.52 |
| Male sex | 1.16 (0.82–1.65) | 0.40 |
| APACHE II score | 1.07 (1.05–1.09) | <0.001 |
| Clinical frailty score | 1.20 (1.08–1.34) | <0.001 |
| Site of infection |  |  |
| Lung | 1.09 (0.70–1.69) | 0.71 |
| Abdomen | 1.10 (0.72–1.69) | 0.66 |
| Urinary tract | 0.47 (0.29–0.77) | 0.003 |
| Others | Reference |  |
| Comorbidity |  |  |
| Chronic heart failure | 0.94 (0.60–1.46) | 0.78 |
| Chronic kidney disease | 1.08 (0.68–1.71) | 0.73 |
| Diabetes | 0.79 (0.54–1.15) | 0.21 |
| Chronic hypertension | 0.93 (0.66–1.31) | 0.68 |

APACHE: Acute Physiology and Chronic Health Evaluation; MAP: mean arterial pressure.

Hazard ratio associated with a one-unit change of age, APACHE II score, and clinical frailty score. Interaction hazard ratios indicate how the effect of the high-MAP target strategy differs from that in the normal-body mass index group.

**Supplementary Table S3.** Cox regression analysis to identify the associations between body mass index categories and mortality in the different blood pressure target groups.

A. Univariate

|  | Control group | | High-target group | |
| --- | --- | --- | --- | --- |
|  | Hazard ratio (95% CI) | P-value | Hazard ratio (95% CI) | P-value |
| Body mass index category |  |  |  |  |
| Underweight | 1.47 (0.83–2.61) | 0.18 | 1.75 (1.06–2.88) | 0.03 |
| Normal | Reference |  | Reference |  |
| Higher BMI | 0.76 (0.42–1.40) | 0.39 | 1.36 (0.82–2.23) | 0.23 |

B. Multivariate

|  | Control group | | High-target group | |
| --- | --- | --- | --- | --- |
|  | Adjusted hazard ratio (95% CI) | P-value | Adjusted hazard ratio (95% CI) | P-value |
| Body mass index category |  |  |  |  |
| Underweight | 1.36 (0.75–2.44) | 0.31 | 1.74 (1.02–2.98) | 0.04 |
| Normal | Reference |  | Reference |  |
| Higher BMI | 0.66 (0.35–1.22) | 0.18 | 1.72 (1.02–2.87) | 0.04 |
| Age | 1.00 (0.97–1.04) | 0.90 | 1.01 (0.98–1.04) | 0.49 |
| Male sex | 1.48 (0.86–2.54) | 0.16 | 1.00 (0.63–1.58) | 1.00 |
| APACHE II score | 1.07 (1.04–1.10) | <0.001 | 1.07 (1.04–1.09) | <0.001 |
| Clinical frailty score | 1.23 (1.04–1.46) | 0.02 | 1.17 (1.03–1.35) | 0.02 |
| Site of infection |  |  |  |  |
| Lung | 0.93 (0.46–1.89) | 0.84 | 1.20 (0.68–2.13) | 0.53 |
| Abdomen | 1.25 (0.63–2.47) | 0.52 | 1.08 (0.63–1.84) | 0.78 |
| Urinary tract | 0.36 (0.16–0.82) | 0.02 | 0.62 (0.33–1.14) | 0.12 |
| Others | Reference |  | Reference |  |

APACHE: Acute Physiology and Chronic Health Evaluation.

Hazard ratio associated with a one-unit change of age, APACHE II score, and clinical frailty score.

**Supplementary Table S4.** Comorbidity-adjusted Cox regression analysis to identify the associations between body mass index categories and mortality in the different blood pressure target groups.

|  | Control group | | High-target group | |
| --- | --- | --- | --- | --- |
|  | Adjusted hazard ratio (95% CI) | P-value | Adjusted hazard ratio (95% CI) | P-value |
| Body mass index category |  |  |  |  |
| Underweight | 1.29 (0.71–2.34) | 0.40 | 1.75 (1.02–3.00) | 0.04 |
| Normal | Reference |  | Reference |  |
| Higher BMI | 0.66 (0.35–1.23) | 0.19 | 1.77 (1.04–3.03) | 0.04 |
| Age | 1.00 (0.97–1.04) | 0.88 | 1.01 (0.98–1.04) | 0.49 |
| Male sex | 1.52 (0.87–2.66) | 0.14 | 1.00 (0.63–1.59) | 1.00 |
| APACHE II score | 1.07 (1.04–1.11) | <0.001 | 1.07 (1.04–1.09) | <0.001 |
| Clinical frailty score | 1.25 (1.05–1.49) | 0.01 | 1.18 (1.03–1.36) | 0.02 |
| Site of infection |  |  |  |  |
| Lung | 0.91 (0.44–1.86) | 0.79 | 1.18 (0.66–2.10) | 0.57 |
| Abdomen | 1.23 (0.61–2.45) | 0.56 | 1.05 (0.61–1.83) | 0.86 |
| Urinary tract | 0.35 (0.15–0.80) | 0.01 | 0.62 (0.33–1.16) | 0.13 |
| Others | Reference |  | Reference |  |
| Comorbidity |  |  |  |  |
| Chronic heart failure | 0.91 (0.45–1.84) | 0.79 | 0.99 (0.56–1.76) | 0.98 |
| Chronic kidney disease | 1.20 (0.58–2.52) | 0.62 | 0.97 (0.53–1.77) | 0.92 |
| Diabetes | 0.72 (0.40–1.29) | 0.27 | 0.82 (0.50–1.33) | 0.41 |
| Chronic hypertension | 0.93 (0.54–1.59) | 0.79 | 0.91 (0.58–1.43) | 0.68 |

APACHE: Acute Physiology and Chronic Health Evaluation.

Hazard ratio associated with a one-unit change of age, APACHE II score, and clinical frailty score.

**Supplementary Table S5.** Cox regression analysis to identify the associations between body mass index categories with a cutoff of ≥25 kg/m² and mortality in the different blood pressure target groups.

A. Univariate

|  | Control group | | High-target group | |
| --- | --- | --- | --- | --- |
|  | Hazard ratio (95% CI) | P-value | Hazard ratio (95% CI) | P-value |
| Body mass index category |  |  |  |  |
| <18.5 | 1.57 (0.92–2.68) | 0.10 | 1.70 (1.06–2.72) | 0.03 |
| 18.5-24.9 | Reference |  | Reference |  |
| ≥25 | 0.79 (0.40–1.57) | 0.50 | 1.42 (0.84–2.40) | 0.19 |

B. Multivariate

|  | Control group | | High-target group | |
| --- | --- | --- | --- | --- |
|  | Adjusted hazard ratio (95% CI) | P-value | Adjusted hazard ratio (95% CI) | P-value |
| Body mass index category |  |  |  |  |
| <18.5 | 1.49 (0.86–2.58) | 0.15 | 1.64 (0.98–2.76) | 0.06 |
| 18.5-24.9 | Reference |  | Reference |  |
| ≥25 | 0.67 (0.33–1.36) | 0.27 | 1.79 (1.04–3.09) | 0.04 |
| Age | 1.00 (0.97–1.04) | 0.90 | 1.01 (0.98–1.05) | 0.37 |
| Male sex | 1.48 (0.86–2.53) | 0.16 | 1.01 (0.64–1.60) | 0.95 |
| APACHE II score | 1.07 (1.04–1.10) | <0.001 | 1.07 (1.04–1.09) | <0.001 |
| Clinical frailty score | 1.23 (1.04–1.46) | 0.01 | 1.16 (1.02–1.33) | 0.03 |
| Site of infection |  |  |  |  |
| Lung | 0.91 (0.45–1.86) | 0.80 | 1.22 (0.69–2.18) | 0.49 |
| Abdomen | 1.23 (0.62–2.44) | 0.55 | 1.09 (0.64–1.87) | 0.74 |
| Urinary tract | 0.36 (0.16–0.82) | 0.01 | 0.65 (0.35–1.20) | 0.17 |
| Others | Reference |  | Reference |  |

APACHE: Acute Physiology and Chronic Health Evaluation.

Hazard ratio associated with a one-unit change of age, APACHE II score, and clinical frailty score.

**Supplementary Table S6.** Trends in the secondary outcomes and adverse events across body mass index categories within each blood pressure target group.

|  | Control group | | |  | High-target group | | |  |
| --- | --- | --- | --- | --- | --- | --- | --- | --- |
|  | Underweight | Normal | Higher BMI | P-value | Underweight | Normal | Higher BMI | P-value |
| 28-day ventilator-free days | 28 (0–28) | 22 (0–28) | 25 (16–28) | 0.85 | 15 (0–27) | 23 (0–28) | 21 (0–28) | 0.60 |
| 28-day RRT-free days | 28 (0–28) | 28 (11–28) | 28 (25–28) | 0.73 | 28 (0–28) | 28 (8–28) | 28 (0–28) | 0.99 |
| 28-day vasopressor-free days | 25 (17–26) | 24 (18–25) | 25 (20–26) | 0.27 | 22 (0–25) | 24 (19–25) | 23 (0–25) | 0.95 |
| Any arrhythmia, n (%) | 5 (7.7) | 10 (11.4) | 9 (9.8) | 0.71 | 9 (14.8) | 12 (11.4) | 7 (9.0) | 0.29 |
| Ischemic events, n (%) | 4 (6.2) | 1 (1.1) | 4 (4.3) | 0.67 | 3 (4.9) | 5 (4.8) | 9 (11.5) | 0.11 |
| Hemorrhagic events, n (%) | 1 (1.5) | 0 (0.0) | 2 (2.2) | 0.62 | 1 (1.6) | 4 (3.8) | 3 (3.8) | 0.49 |

RRT, renal replacement therapy.

Values are expressed as median (interquartile range) or n (%). P-values were calculated using Cochran-Armitage test or Jonckheere-Terpstra test.

**Supplementary Table S7.** Trends in the management required to maintain blood pressure during the 72 hours after randomization across body mass index categories within each blood pressure target group.

|  | Control | | |  | High-target | | |  |
| --- | --- | --- | --- | --- | --- | --- | --- | --- |
|  | Underweight | Normal | Higher BMI | P-value | Underweight | Normal | Higher BMI | P-value |
| Fluid volume, mL | 6,504 (5,100–8,096) | 7,395 (5,400–9,886) | 6,833 (4,662–9,911) | 0.48 | 6,655 (4,865–8,680) | 6,699 (4,853–8,752) | 7,262 (4,936–10,087) | 0.55 |
| Fluid volume/BW, mL/kg | 156 (123–211) | 137 (97–208) | 116 (69–155) | <0.001 | 167 (121–203) | 127 (93–169) | 109 (74–151) | <0.001 |
| Norepinephrine, mg | 9.75 (4.10–21.42) | 9.42 (4.94–22.32) | 10.60 (5.26–24.50) | 0.32 | 14.50 (7.55–22.34) | 15.30 (5.80–25.73) | 17.10 (7.15–32.60) | 0.17 |
| Norepinephrine/BW, mg/kg | 0.26 (0.10–0.50) | 0.18 (0.10–0.37) | 0.15 (0.08–0.37) | 0.14 | 0.39 (0.17–0.56) | 0.31 (0.12–0.50) | 0.29 (0.11–0.48) | 0.39 |
| Vasopressin, unit | 1.99 (0.00–31.30) | 12.10 (0.00–49.00) | 18.10 (0.00–57.04) | 0.01 | 33.88 (9.28–68.73) | 35.40 (5.73–64.30) | 37.00 (11.00–80.50) | 0.71 |
| Vasopressin/BW, unit/kg | 0.06 (0.00–0.82) | 0.24 (0.00–1.02) | 0.32 (0.00–0.82) | 0.19 | 0.84 (0.21–1.68) | 0.66 (0.10–1.21) | 0.67 (0.18–1.22) | 0.23 |
| Epinephrine, mg | 0.0 (0.0–0.0) | 0.0 (0.0–0.0) | 0.0 (0.0–0.0) | 0.61 | 0.0 (0.0–0.0) | 0.0 (0.0–0.0) | 0.0 (0.0–0.0) | 0.38 |
| Epinephrine/BW, mg/kg | 0.0 (0.0–0.0) | 0.0 (0.0–0.0) | 0.0 (0.0–0.0) | 0.61 | 0.0 (0.0–0.0) | 0.0 (0.0–0.0) | 0.0 (0.0–0.0) | 0.39 |

BW, body weight.

Values are expressed as median (interquartile range). P-values were calculated using Jonckheere-Terpstra test.

**Supplementary Figure S1.** Patient flow diagram.

BMI, body mass index.

**Supplementary Figure S2.** Survival curves between body mass index categories in the control and high-target groups.

The control group showed a significant trend toward lower mortality across increasing body mass index categories. Higher-BMI patients had higher mortality than those with normal body mass index in the high-target group.

P-values were calculated with Log-rank test for trend.

**Supplementary Figure S3.** 90-day mortality in the control and high-target groups using a cutoff of ≥25 kg/m² to define the higher body mass index category.

Patients in the control group showed a significant inverse trend in mortality across increasing BMI categories (P=0.03). Patients in the high-target group had the highest mortality among underweight patients, and patients with a BMI ≥25 kg/m² also exhibited high mortality, with no significant trend across BMI categories (P=0.28).

P-values were calculated with Cochran-Armitage test for trend.

**Supplementary Figure S4.** Survival curves in the control and high-target groups using ≥25 kg/m² to define the higher body mass index category.

The control group showed a significant trend toward lower mortality across increasing BMI categories. In the high-target group, patients with a BMI ≥25 kg/m² had higher mortality than those with a BMI of 18.5–24.9 kg/m².

P values were calculated using the log-rank test for trend. BMI, body mass index.

**Supplementary Figure S5.** Blood pressure management according to mean arterial pressure target across different body mass index categories.

Data are presented as medians with interquartile ranges. Baseline values are shown descriptively and were excluded from the mixed-effects analyses. P values for BMI category and the BMI category-by-time interaction were obtained from mixed-effects models fitted by restricted maximum likelihood using measurements from 4 to 72 hours.
